# The rise and fall of Australian physical activity policy 1996 – 2006: a national review framed in an international context

**DOI:** 10.1186/1743-8462-5-18

**Published:** 2008-07-31

**Authors:** Bill Bellew, Stephanie Schöeppe, Fiona C Bull, Adrian Bauman

**Affiliations:** 1 School of Public Health, University of Sydney, Sydney, Australia; 2 Centre for Physical Activity and Health, Building K25, University of Sydney, Sydney, Australia; 3 School of Sport and Exercise Science and BHF National Centre on Physical Activity and Health, Loughborough University, Loughborough, UK

## Abstract

**Background:**

This paper provides an historical review of physical activity policy development in Australia for a period spanning a decade since the release of the US Surgeon General's Report on Physical Activity and Health in 1996 and including the 2004 WHO Global Strategy on Diet, Physical Activity and Health. Using our definition of '*HARDWIRED*' policy criteria, this Australian review is compared with an international perspective of countries with established national physical activity policies and strategies (New Zealand, Canada, Brazil, Scotland, Switzerland, the Netherlands and Finland). Methods comprised a literature and policy review, audit of relevant web sites, document searches and surveys of international stakeholders.

**Results:**

All these selected countries embraced multi-strategic policies and undertook monitoring of physical activity through national surveys. Few committed to policy of more than three years duration and none undertook systematic evaluation of national policy implementation. This Australian review highlights phases of innovation and leadership in physical activity-related policy, as well as periods of stagnation and decline; early efforts were amongst the best in the world but by the mid-point of this review (the year 2000), promising attempts towards development of a national intersectoral policy framework were thwarted by reforms in the Federal Sport and Recreation sector. Several well received reviews of evidence on good practices in physical activity and public health were produced in the period but leadership and resources were lacking to implement the policies and programs indicated. Latterly, widespread publicity and greatly increased public and political interest in chronic disease prevention, (especially in obesity and type 2 diabetes) have dominated the framework within which Australian policy deliberations have occurred. Finally, a national physical activity policy framework for the Health sector emerged, but not as a policy vision that was inclusive of the other essential sectors such as Education, Transport, Urban Planning as well as Sport and Recreation.

**Conclusion:**

Despite some progression of physical activity policy in the decade since 1995/6, this review found inconsistent policy development, both in Australia and elsewhere. Arguably, Australia has done no worse than other countries, but more effective responses to physical inactivity in populations can be built only on sustainable multi-sectoral public health policy partnerships that are well informed by evidence of effectiveness and good practice. In Australia and elsewhere prerequisites for success are political support, long-term investment and commitment to program implementation and evaluation. An urgent priority is media and political advocacy for physical activity focussed on these factors.

## Introduction

Policy approaches to promoting physical activity at the population level are a central component of addressing increasing global rates of non-communicable diseases^1 ^[1]. Physical activity policy has been defined as:

"*a formal statement that defines physical activity as a priority area, states specific population targets and provides a specific plan or framework for action." Further, it is held that a policy should 'describe the procedures of institutions in the government, non government and private sector to promote physical activity in the population, and defines the accountabilities of the involved partners*"[2].

The characteristics of successful physical activity policy have been drawn from a sparse literature and from recent international consensus meetings where experiences from around the world have been shared [2,3]. These emerging criteria for a successful policy process and product are set out here in a newly revised and condensed form under the descriptive label ***HARDWIRED ***– reflecting our view that these are the characteristics absolutely essential for national physical activity policy development and need to be embedded for the long-term in order to deliver successful outcomes. The criteria (expanded in Table 1) are that national physical activity policy be:

**Table 1:** Criteria for successful national physical activity policy [*HARDWIRED*]

***1. Highly consultative in development***
Thorough stakeholder analysis and needs assessment is used to determine and drive appropriate consultations at an early stage and during the policy development process; it engages 'grassroots' practitioners as well as strategic policymakers, and defines their organisational linkages and relevance to the physical activity agenda;
***2. Active through multi-strategic, multi-level, partnerships***
Progression of policy through coalitions and partnerships (e.g. across government sectors, non government agencies as well as the private sector); a comprehensive approach using multiple strategies (individual-oriented behaviour change, environmental-focused interventions, mass media campaigns) at multiple levels (local, state, national level) and targeting multiple population groups (e.g. children, adolescents, women, older adults, disabled people, indigenous people);
***3. Resourced adequately***
There is a stable base of political and stakeholder support, an adequate capacity to implement strategies across the sectors as well as an adequate, sustained investment to implement the policy over the long term;
***4. Developed in stand-alone and synergistic policy modes***
A clear 'stand-alone'/single issue physical activity policy statement is developed accompanied by several related strands of physical activity policy embedded within other related agendas (e.g. in the fields of health, nutrition and obesity, education, transport, urban planning, greenhouse energy management) to achieve synergistic policy impacts;
***5. Widely communicated***
Clear identification and communication of the policy is achieved through marketing and by tailoring of communication styles to match a specific market segmentation (e.g. politicians, senior bureaucrats, researchers, community based practitioners, general public);
***6. Independently evaluated***
There is a specific plan to evaluate the implementation (process), impact (short term results) and outcomes (longer term results) of the policy; the evaluation is ideally conducted independently of government and of the policy 'owners';
***7. Role-clarified and performance-delineated***
Roles and responsibilities of agencies involved in policy implementation are well clarified (e.g. lead agency, supporting agency, consulting agency) and there is common understanding of and agreement on how 'successful implementation' is to be defined and measured (e.g. 'smart' performance indicators incorporating measurable targets, achievement criteria, timeframes);
***8. Evidence-informed and Evidence-generating***
Systematic surveillance of population physical activity; evaluation of innovative programs; policy-relevant syntheses of epidemiological and other relevant evidence (e.g. trends, priority populations, activity preferences, evaluation findings) disseminated in formats accessible by the target audiences; and
***9. Defined national guidelines for health enhancing physical activity***
Dissemination of National guidelines for health enhancing physical activity that are developmentally and age-appropriate (e.g. children and adolescents, adults, older adults). It may also seek to define physical activity guidelines in relation to specific diseases and conditions (e.g. for the management/prevention of type 2 diabetes, or for the prevention of certain cancers and cardiovascular disease). These detailed 'prescriptions' lend themselves to individual communication and typically in the primary care setting; specific (e.g. Cancer, Heart, Diabetes) non-government organisations can play a useful role in leading the production of this guidance.

**H**ighly consultative in development;

**A**ctive through multi-strategic, multi-level, partnerships;

**R**esourced adequately;

**D**eveloped in stand-alone and synergistic policy modes;

**W**idely communicated;

**I**ndependently evaluated;

**R**ole-clarified and performance-delineated;

**E**vidence-informed and Evidence-generating; and

**D**efined national guidelines for health enhancing physical activity.

Using this definition and the criteria outlined, we trace the history of physical activity policy development in Australia over the decade 1995/6–2005/6 and present our discussion in the context of the results of an international physical activity policy scan conducted during 2003 and 2004.

## Methods

### (a) International review of national physical activity policy

A literature and policy review was undertaken for seven selected countries (New Zealand, Canada, Brazil, Scotland, Switzerland, Netherlands, and Finland) for which there were evidence of large scale or national policy on physical activity. For these countries, government and non-government web-sites were audited to identify any formal policy statements on physical activity (including policy documents, strategic plans or frameworks for action) as well as any supporting documents or reports. Any statements consistent with our definition of physical activity policy were sought and all materials were assessed against defined criteria [2,4]. To further supplement the information found in written documents, we conducted an electronic survey of key international physical activity and public health stakeholders and experts in these countries to gain further insight in to the policy development and implementation process. Criteria for selecting experts for participation in the survey were that the survey respondent:

(i) worked in or closely with government and was responsible for or close to the work on physical activity policy (development, coordination, implementation);

(ii) worked in physical activity and public health research with experience and close affiliation to government; and

(iii) was nationally recognised and experienced in physical activity policy (development, coordination and implementation).

In total 8 experts responded to the electronic questionnaire with responses obtained from six countries (Australia, New Zealand, Brazil, Scotland, the Netherlands and Finland) with a national agenda on physical activity and two responses from countries which at the time did not have a current national policy but did have a history of significant development attempts (England and the USA).

Questionnaires were completed electronically and analysed in Microsoft Excel. Frequencies of close-coded response categories were computed and text responses were collated. The international physical activity policy search and electronic survey was conducted over the period August 2003 to June 2004. Findings of the international study (alone) have been previously summarised but without any detailed comparative analysis to concurrent Australian policy developments [2,4]. The key findings of the international review of national physical activity policy are summarised in Table 2 (refer also to Table 4).

**Table 2:** Key findings of the international physical activity policy scan

***All countries***
• Undertook broad consultation with key stakeholders from different sectors;
• Attempted to integrate physical activity policy with other national policy agendas;
• Incorporated multiple strategies (particularly multiple individual-oriented components and to a lesser extent, environmentally-focused interventions);
• Worked (or planned to work) at multiple levels (e.g., national, state, local) to coordinate and implement their policies; and
• Achieved some monitoring of population levels of physical activity through national surveys.
***Some countries***
• Initiated the development of coalitions and partnerships within and between governmental and non governmental organisations, and in some cases also involving the private sector;
• Developed a clear identity or branding for the initiative; and
• Developed national physical activity guidelines targeted to the general adult population.

***Few countries***
• Established clear delineation of responsibilities for coalition members for specific strategy components;
• Indicated clear timeframes for funding;
• Gave a time commitment to policy greater than three years duration;
• Could provide information on current practice and programs;
• Could articulate specific activities planned for implementation in the near future; and
• Were able to maintain physical activity policy initiatives for more than a few years.

***No countries***
• Established a systematic approach to monitoring and evaluating the implementation of the physical activity policy.

### (b) Historical review of Australian physical activity policy

Authors (BB, AB and FB) were actively involved in physical activity policy and research across government and non-government agencies at Federal and State levels during the period of the Australian historical review (1995/6–2005/6). Paper and electronic records for the review period were also examined by BB who was a State government officer and inaugural chair of the Strategic Inter-Governmental forum on Physical Activity and Health (SIGPAH), by AB who was a University academic and inaugural chair of Australia's first Task Force on Physical Activity and by FB who was a university academic and chair of the SIGPAH working party on general practice. The methods of this review comprised compiling a catalogue of events and policy development in Australia in historical sequence; this was written, checked by co-authors and refined in consultation with key government department managers appointed during the review period [see acknowledgments].

#### Physical Activity Policy Development in Australia 1996 – 2006

The decade of physical activity policy development in Australia from 1995/6 included periods of innovation and leadership, periods of progress and periods of stagnation and decline. Important dates and corresponding policy initiatives or events are shown in Table 3.

**Table 3:** Key physical activity policy events in Australia^+ ^1995/6 – 2005/6

**1995**	• USA: Physical Activity and Public Health – A Recommendation from CDC and ACSM
	• Physical Activity and Health: a special communication from the NSW Chief Health Officer'
	• Active and inactive Australians' research report published
**1996**	• Towards best practice for physical activity in the areas of NSW
	• USA: Physical activity and health: a report of the Surgeon General
	• NSW Physical Activity Task Force established by Premier, launched by Deputy Premier
	• Active Australia concept commences

**1997**	• Acting on Australia's weight: a strategic plan for the prevention of overweight and obesity published
	• Active Australia – A National Participation Framework launched by Federal Ministers
	• First Active Australia Survey conducted

**1998**	• First Active Australia Campaign (25–60 yrs) implemented in NSW February-March
	• Developing an Active Australia: A framework for action for physical activity and health
	• launched by Federal Health Minister

**1999**	• Active Australia Media Campaign (older adults targeted, 55–75 years)
	• SIGPAH inaugural meeting in Canberra 6–7 May
	• National Physical Activity Guidelines for adults released by Australian Government (May)
	• Burden of Disease and Injury in Australia published by AIHW (November)
	• Active Australia Alliance established – national level intersectoral planning

**2000**	• Review of Active Australia/consultation for Backing Australia's Sporting Ability
	• Endorsement of Active Australia Alliance National Plan 2000–2003 deferred
	• The Costs of Illness Attributable to Physical Inactivity in Australia published (July) [2000]

**2001**	• Backing Australia's Sporting Ability released by Prime Minister (April) [2001]

**2002**	• Getting Australia Active: towards better practice for the promotion of physical activity (review of evidence) published (March)
	• National Obesity Taskforce established (November

**2003**	• Healthy Weight 2008 endorsed by Australian Health Ministers (November

**2004**	• Australian Prime Minister announces $116M funding package over 4 years to implement
	• *Building a Healthy Active Australia *initiative (June) [2004]
	• National physical activity recommendations for 5–12 and 12–18 year olds (July)
	• WHO Global Strategy on Diet, Physical and Health (DPAS) released

**2005**	• Physical activity guide for older Australians released (April)
	• Be Active Australia: A Framework for Health Sector Action for Physical Activity 2005 -
	• 2010 (July)
	• National Chronic Disease Strategy endorsed by Australian Health Ministers (November)

**2006**	• Australian Government publication of Healthy Weight for Adults and Older Australians – a
	• national action agenda to address overweight and obesity in adults and older Australians
	• AusPANet established as an independent initiative mid 2006 under the auspices of the National Heart Foundation of Australia and the University of Sydney; AusPANet. was set up to build knowledge and capacity in the physical activity workforce (website, fortnightly e-News bulletin, 'ask an expert' function [from 2007]

^+ ^some key international events included because of impact in Australia.

NSW: New South Wales ; AIHW: Australian Institute of Health and Welfare; AusPANet: Australian Physical Activity Network

**Table 4:** Australian physical activity policy development reviewed against *HARDWIRED *criteria and from an international perspective

***HARDWIRED *Criteria for National Policy**	**Countries studied in International Review**	**Australia**
**Highly consultative in development;**	++++	+++
**Active through multi-strategic, multi-level, partnerships;**	+++	+++
**Resourced adequately;**	++	+
**Developed in stand-alone and synergistic policy modes;**	+++	++
**Widely communicated;**	+++	+++
**Independently evaluated;**	0	0
**Role-clarified and performance-delineated;**	+	++
**Evidence-informed and Evidence-generating; and**	+++	++
**Defined national guidelines for health enhancing physical activity**.	+++	++++

KEY

++++ = Substantially achieved

+++ = Partially achieved

++ = Partial progress

+ = Little progress

0 = No progress

An initial stimulus was in 1995 when the Chief Health Officer of New South Wales (NSW, Australia's most populous State) issued a Special Communication on Physical Activity and Health[5] This policy communiqué closely followed the special communication from the Centers for Disease Control (CDC)/American College of Sports Medicine (ACSM) in January of that year [6] recommending that "Every adult in New South Wales should accumulate 30 minutes of moderate-intensity physical activity on most, preferably all, days of the week." Research on the physical activity preferences of Australians was released in that same year provided important information and strategic principles for policymakers [7]. The State of Victoria had the benefit of a social marketing campaign 'Active for Life" which incorporated community walking events, links with local government and a telephone information line. The initiative was developed by the Department of Human Services with the support of Victorian Health Promotion Foundation, VicFit and the Heart Foundation. Policy development was given further impetus the following year when the NSW State Health Department published an evidence based guide to the promotion of physical activity [8]. Although produced at State level, the publication was requested by governments and NGOs throughout Australia. This so-called 'red book' was published under the auspices of the NSW Premier's Physical Activity Task Force (PATF) an intersectoral body given responsibility by the NSW State government to promote physical activity to the people of NSW. These initial developments were reinforced by the landmark publication of the United States Surgeon General Report Physical Activity and Health and by its attendant publicity [9].

From 1995 (the year in which Sydney secured the millennium Olympic Games), the Australian Sports Commission (ASC – a Federal agency concerned mainly with sporting excellence) broadened its policy agenda to include community participation in physical activity. As a result, the government Sport and Recreation departments across Australia's States and Territories agreed to work with the ASC to develop a national framework. This heralded the formation of Active Australia as a truly national policy framework and paved the way for fertile policy discussion and partnerships. Initial partners were the (Federal level) Australian Sports Commission and Commonwealth Department of Health (DoH) and the (State level) NSW Physical Activity Task Force. The focus of the policy framework was on health enhancing (including incidental) physical activity within a broadened definition of physical activity. This consensus definition of physical activity spanned organised and elite forms of sport and recreation participation as well as less structured forms and transport-related physical activity and provided a platform for collaboration across policies and social marketing programs under the common "Active Australia" brand.

Three main streams of activity ensued – (i) intersectoral policy development and implementation; (ii) social marketing campaigns; and (ii) initial development of national surveillance systems for physical activity. Joint core funding was provided by the ASC, DoH and the NSWHealth Department for a pilot public education campaign in NSW featuring the slogan "Exercise. You only have to take it regularly not seriously" [10]. The collaboration established to support campaign implementation non Government organisations, State Departments of Education, Sport & Recreation, private sector Fitness Industry bodies, as well as the three core funding agencies. The Active Australia Survey was first developed and nationally implemented in 1997 to assess the effectiveness of the campaign in NSW and provide national physical activity prevalence data. The original survey methodology was subsequently implemented nationally through the National Physical Activity Surveys in 1999 and 2000 and through the Australian Diabetes, Obesity and Lifestyle Study in 1999–2000. It was also used in various formats in State-based surveys, such as in Queensland, South Australia, New South Wales and Western Australia [11,12]. This provided a better approach to national physical activity surveillance.

In July 1997, Active Australia–a national participation framework was formally endorsed by the Federal ministers for Sport and Health and all States' and Territories' ministers for Sport [13]. A National Symposium in February 1997 and a Workshop in October that year formulated the Health sector's more specific contribution to Active Australia; this was released as a policy statement and a framework for action by the Federal Health Minister in June 1998[14]. This was the first National Physical Activity policy statement for Australia, and included specific strategies in (i) public education, (ii) physical environments, (iii) infrastructure and capacity building and (iv) monitoring. At the state level, the NSW PA Task Force released its 5-year strategic plan for physical activity in 1998; the introduction section described formal acknowledgment by the World Health Organisation that this had been a model of best practice in health promotion [15]. March of that same year saw a second phase of the social marketing campaign activity in NSW targeting older Australians and designed to coincide with the United Nations International Year of Older Persons [16].

A national peak physical activity policy group was developed within the Health sector; this was the Strategic Inter-Governmental Forum on Physical Activity and Health (SIGPAH). It was established in 1999 with Government health representatives from all Australian States and Territories as well as observers from the Australian Institute of Health and Welfare (AIHW) and the Australian Sports Commission (ASC) [17]. By mid-year 1999 the Federal (Commonwealth) Health Department released the first National Physical Activity Guidelines for adults [18]. It is interesting, given the subsequent prominence of obesity-related policy, to note that the policy imprimatur and consequently the funding allocation for the 1999 adult physical activity guidelines came out of an earlier obesity policy context (Acting on Australia's weight)[19]. In November 1999 the AIHW published the Burden of Disease and Injury in Australia study [20]. The study was important for physical activity policy because it highlighted that in terms of preventable risk factors, physical inactivity ranked second after tobacco, and made twice the contribution of obesity, to overall morbidity and mortality in Australia.

By the end of 1999 a new national committee, known as the Active Australia Alliance (the Alliance), was established to formalise an intersectoral policy approach between Sport, Recreation and Health and to oversee the implementation and monitoring of Active Australia. The Alliance developed a draft National Plan 2000–2003 through a robust consultation process to a point where it had the endorsement of all key constituencies, was cross linked with other important plans (such as the work plan developed by SIGPAH) and was ready for formal approval and implementation. This planning process and resultant policy document represented the first comprehensive intersectoral policy for physical activity at national level in Australia. Unfortunately, this draft policy document did not receive the necessary approval from government. The timing coincided with the Federal Government's decision to change its stance on the role of ASC – the national Sport and Recreation agency, within broad physical activity policy. The government embarked on a review, and subsequently a shift in focus of the ASC. This was a policy shift by the Sports Commission, away from community participation. This changed the Active Australia initiative, removing its comprehensive focus and interagency partnerships, and undermining physical activity policy progress that had been achieved in the previous four years.

The elite sport orientation was further developed through a new sport policy, Backing Australia's Sporting Ability [21]. The announcement came with a large government funding package reorienting Active Australia and the services performed by the Australian Sports Commission to focus on participation in elite sport. This limited the opportunities for collaboration with the Health sector. The Active Australia Alliance National Plan 2000–2003 was shelved and, given its demise, all cross referencing to it was duly removed from its new work plan by SIGPAH [22].

As if to underscore the magnitude of the lost opportunity, a new report on the costs of illness attributable to physical inactivity in Australia was published in July 2000 [23]. The report provided direct cost of illness estimates for physical activity for the first time; whilst these estimates were conservative, they nonetheless indicated costs of at least $377 million per year.

The subsequent period 2002–2005 was characterised by rapid and fragmented change. National guidelines towards better practice in the promotion of physical activity were published in 2002 [24]; but physical activity policy levers were no longer present. There was also a remarkable and sudden rise in public and political interest in the issue of childhood obesity, leading ultimately to the establishment of a high level National Obesity Taskforce (NOTF). As a consequence of NOTF's work the following year saw the related release of Healthy Weight 2008 [25] (a national policy initiative on overweight and obesity with a focus on childhood) and then in 2004, the Prime Minister announced $116 million of funding over four years for the Building a Healthy Active Australia initiative to address childhood obesity through an after-schools program to tackle declining physical activity and poor eating habits of Australian children [26]. Notwithstanding the high profile they achieved, many of the early policy steps on obesity fitted with existing implementation systems within State and Federal governments. This reflected an incremental approach to policy formulation rather a more strategic platform, but may have built sufficient commitment and support to create momentum for more strategic policy at a later time [27].

Further documents were released in 2004, including physical activity recommendations for children and adolescents and older adults [28]. In this year, the World Health Organisation (WHO) released its Global Strategy on Diet, Physical Activity and Health (DPAS) [1] that physical activity on the global NCD prevention and public health agenda. A revised Health-sector-specific national framework Be Active Australia: A Framework for Health Sector Action for Physical Activity 2005–2010 [29] were released in the course of 2005. However, these documents had no direct national policy connections, and demonstrated the need for guidelines and strategic plans to be linked to active policy effector mechanisms.

The last few years of the decade under review saw increased government emphasis on chronic disease prevention – epitomised by the National Chronic Disease Strategy which was endorsed by Australian Health Ministers in 2005 [30]. The integrated approach was reflected in organisational arrangements to support key strategy groups, and SIGPAH was redesignated as a "Communication Network" under the Chronic Disease and Injury Prevention Working Group. In effect, this was the demise of SIGPAH as originally constituted, and the marginalization of physical activity as a 'supportive role', subservient to the stronger focus on policy developments in obesity and diabetes [31].

## Discussion

This paper describes Australia's track record in physical activity policy development in an international context; Table 4 summarises the analysis. Internationally, all countries studied undertook broad consultation with key stakeholders from different sectors; Australia did so at the State level but failed to deliver adequately on this criterion at the national level. All countries worked (or intended to) at multiple levels (Local, State, Federal); Australia succeeded in doing this quite well at State but not at the Federal level. SIGPAH provided useful coordination between Federal and State levels in the Health sector and for a period this coordination also extended to the Sport and Recreation sector; but this effort was not sustained.

In our view, no countries adequately resourced the implementation of national physical activity policy. There were attempts overseas to integrate physical activity policy with other national agendas (more so with nutrition and obesity than with transport and environment) for synergistic effects; Australia followed this pattern with the release of national policy frameworks on obesity which were designed to take account of State level initiatives and policies in physical activity but ended up absorbing physical activity policy initiatives without providing the investment required to implement them.

Some countries used a brand or clear identity for national policy; good examples were the 'Agita' program in South America [32] and ParticipACTION in Canada [33,34]. The Active Australia brand (especially in the period 1996–1999) had provided a powerful unifying social marketing brand for the promotion of the whole spectrum of physical activities from organised sport through to less structured forms of physical activity. It also provided an organisational structure and communication networks for linkages and partnering at Federal and State levels, between the sectors of Health, Sport & Recreation and others (e.g. education, and NGOs such as the National Heart Foundation of Australia). This structure provided a potential mechanism for shared information, shared resources and shared problem-solving for physical activity policy development and implementation. However, as noted, this brand of policy was ultimately re-labelled through redefinition of priorities and reorganisation towards one that did not include broader approaches to health-enhancing physical activity at the population level but was predominantly focused on elite sport.

Neither overseas nor in Australia was there evidence of success at national level in the clear delineation of coalition roles and responsibilities, matched with long term commitment, or evaluation of policy implementation. Surveillance was one areas that has been approached particularly poorly. All countries studied achieved some monitoring of population levels of physical activity through national health or physical activity surveys, but surveys were erratic and questions were changed, making prevalence trends difficult to discern. Exceptions to this were in Finland and in Canada, where standardized long term surveillance was effectively maintained [35-37] Australia achieved only a piecemeal and ultimately deficient effort at systematic national PA surveillance. One area of success was in the development of national physical activity guidelines in Australia tailored to adults (1999), older adults (2005) as well as children and young people (2004); Canada was one of very few countries in the international study to match this success.

The decade under study saw physical activity emerge from being the "new kid on the block" to being transiently accepted as one of the most important mainstream factors affecting public health. Surveillance data were sometimes used effectively for policy advocacy but less so for the monitoring of policy implementation. Capacity for physical activity policy initially grew so that by the time SIGPAH was formally established (1999) it was able to include representatives from every part of Australia, with most being specialists in physical activity. Australia experimented with and became more sophisticated at comprehensive and intersectoral approaches to physical activity policy. Whilst almost two years were spent in its development phase (1996–98), it is arguable that the NSW Physical Activity Task Force (PATF) had a basis for action well ahead of the release of the formal policy documentation, that it planted the seeds of intersectoral policy development in Australia and may have encouraged accelerated growth of this form of policy in other jurisdictions and countries through a willingness to share "warts 'n all" information and experiences. The policy changes in the national Sport sector, and policy reorientation towards obesity came to overshadow national physical activity developments. By the end of the period, national physical activity policy had a subservient rather than stand-alone expression (through obesity and diabetes). The latter however was exclusively Health sector focussed and in our view too narrow to achieve the desired outcomes that would result from optimal interagency partnerships around physical activity policy.

Initially, Active Australia had been a catalyst for physical activity policy and for the establishment of strong communication networks across State and sector domains. Notwithstanding the demise of Active Australia, some of this capacity and infrastructure appears to have survived at the State level. Western Australia launched its own State level Task Force mid-2001 with high level support from the State government; it went on to become one of the most effective Task Force in Australia [38]. Recent developments in communications have given rise to an independent national physical activity network, AusPANet that provides a rapid information sharing and evidence-clearinghouse function [39]. On the Global stage the very existence of the WHO Global Strategy on Diet, Physical Activity and Health (DPAS) is encouraging, although almost ignored within the Australian policy context. Nonetheless, it has fostered international physical activity network development, both in the Americas [40] and in the Asia-Pacific region (the Asia Pacific Physical activity network, launched in 2006) [41]. These may prove to be useful regional catalysts for the implementation of DPAS.

Limitations of this review include the narrow time focus (2003/4) of the international policy study and fact that it did not attempt to cover all physical activity policies worldwide but rather provided a selection of countries that had recognised physical activity policies at national level, and for which information was available in English or in German. The international experts' appraisal of physical activity policy supported our perception of what has been achieved and where the gaps lay; but we acknowledge the limitations of our methodology (a questionnaire administered to a small selected group of experts) in providing in-depth information on policy implementation. The national historical review covers objective historical facts but is likely also to reflect the personal perspectives of the authors, three of whom were involved in physical activity policy and research in the decade under discussion.

## Conclusion

Our definition of physical activity and our *HARDWIRED *criteria for successful physical activity policy may be useful for those engaged in development and assessment of such policies. An effective public health response to physical inactivity needs to be underpinned by sustainable multi-sectoral public health policy. Available good practice examples and guidelines for strategy development could usefully be examined as a first step by countries or regions in the process of considering physical activity policy or strategy development [42-44].

Despite progression of physical activity policy in the decade since 1995/6 this review found less than the optimal conditions, both globally and in Australia, that might inspire confidence of progress in the decade ahead. In Australia, prerequisites for success are political support, long-term investment and commitment to the evaluation of policies and programs implemented. An urgent priority is media and political advocacy focussed on these factors.

## Competing interests

The authors declare that they have no competing interests.

## Authors' contributions

BB undertook the Australian historical review and was main author of this paper; FB supervised the international review, contributed to the development, drafting and editing of the paper; SS undertook the international review and contributed to early drafts; AB supervised this work and provided extensive comments on drafts of the manuscript.
